# 

**Published:** 1909-07-31

**Authors:** 


### ANNUAL REPORT
### OF THE COMMITTEE OF DISTRIBUTION TO THE COUNCIL.

The Executors of the late Mr. George Herring will pay to the Treasurer of this Fund on July 27, £24,000, being the balance of income available from the Estate, which, with £1,352 received in dividends, etc., upon Securities transferred to the Fund, makes the total income from this source to date £25,352.

The Committee recommend that this sum be added to the collections, making the total of the Fund to date £67,951, in which is included £2,000 the balance of the Legacy of £10,000 under the Will of the late Mr. Herbert Lloyd, £900 being the Legacy, less duty, of Edwin Gayford, deceased, and £500 on account of the Legacy of the late Mr. C. E. Layton.

This year 258 Institutions have applied for grants from the Fund, being one less than in 1908. Two Institutions were considered ineligible. The Committee, while fully realising the excellent work carried on by the Cancer Hospital, have not made an award this year, for the reason stated in their report of 1906.

The Committee do not recommend awards to the Throat Hospital, Golden Square, or the Evelina Hospital, this year, as they do not appear to the Committee to be in need of funds.

The Committee now recommend the distribution of £67,212 to the 164 Hospitals and Institutions, 59 Dispensaries and 30 Nursing Associations shown in the Appendix hereto.

The Committee having reduced the bases of award in 17 cases, the fact was intimated to each of the Hospitals interested in accordance with Law V. Six deputations attended the Committee, and after hearing their several explanations the final awards were determined. Five per cent. of the total sum available for distribution is appropriated to the purchase of surgical appliances during the ensuing year, and 2½ per cent. for district nursing associations.

In compliance with an order of the Council, statistics have been prepared as usual for the special use of its members, showing the number of beds in Hospitals, the cost of patients at Hospitals and Dispensaries, the proportionate expense of management to maintenance, and other valuable information.

A copy thereof has been sent to each Member of the Council.

George Wyatt Truscott,
Lord Mayor, President and Treasurer.

F. A. Bevan. Joseph C. Dimsdale.

William S. Church. Alfred Willett.

Savile Crossley. F. H. Norman.

Stamford. Robert Grey.

Mansion House, E.C., July 19.

### The Awards for 1909 and 1908 Compared.

The following is a complete list of the awards recommended by the Committee of Distribution for the year 1909, compared with those for 1908:

THIRTY-THREE GENERAL HOSPITALS

| | Awards 1909 £ | s. | d. | 1908 £ | s. | d |
|---|---|---|---|---|---|---|
| Charing Cross Hospital | 1,328 | 3 | 4 | 1,530 | 0 | |
| French Hospital | 385 | 13 | 4 | 399 | 7 | 6 |
| German Hospital | 709 | 11 | 8 | 708 | 15 | 0 |
| Great Northern Central Hospital | 1,272 | 18 | 4 | 1,260 | 0 | 0 |
| Guy's Hospital | 1,679 | 3 | 4 | 1,575 | 0 | 0 |
| Hampstead General Hospital | 449 | 11 | 8 | 376 | 17 | 6 |
| Italian Hospital | 218 | 16 | 8 | 225 | 0 | 0 |
| Kensington General Hospital | Nil. | | | 247 | 10 | 0 |
| King's College Hospital | 1,744 | 3 | 4 | 1,800 | 0 | 0 |
| London Hospital | 6,500 | 0 | 0 | 6,300 | 0 | 0 |
| London Homœopathic Hospital | 473 | 8 | 4 | 495 | 0 | 0 |
| Phillips' Memorial Homœopathic Hospital | 42 | 5 | 0 | 51 | 15 | 0 |
| London Temperance Hospital | 748 | 11 | 8 | 776 | 5 | 0 |
| Metropolitan Hospital | 1,022 | 13 | 4 | 1,125 | 0 | 0 |
| Mildmay Hospital | 305 | 10 | 0 | 337 | 10 | 0 |
| Miller Hospital and Royal Kent Dispensary | 276 | 5 | 0 | 270 | 0 | 0 |
| North-West London Hospital | 310 | 18 | 4 | 416 | 5 | 9 |
| Poplar Hospital | 619 | 13 | 4 | 545 | 12 | 6 |
| Prince of Wales's General Hospital, Tottenham | 877 | 10 | 0 | 736 | 17 | 6 |
| Royal Free Hospital | 1,457 | 1 | 8 | 1,462 | 10 | 0 |
| St. George's Hospital | 198 | 5 | 0 | 562 | 10 | 0 |
| S.S. John and Elizabeth Hospital | 203 | 13 | 4 | 371 | 5 | 0 |
| St. John's Hospital, Lewisham | 170 | 1 | 8 | 196 | 17 | 6 |
| St. Mary's Hospital | 2,551 | 5 | 0 | 2,610 | 0 | 0 |
| St. Thomas's Hospital | 208 | 0 | 0 | 168 | 15 | 0 |
| Seamen's Hospital Society | 1,703 | 0 | 0 | 1,665 | 0 | 0 |
| The National Anti-Vivisection Hospital | 97 | 10 | 0 | .. | | |
| The Middlesex Hospital and Convalescent Home | 2,784 | 3 | 4 | 2,700 | 0 | 0 |
| University College Hospital | 2,545 | 16 | 8 | 2,553 | 15 | 0 |
| Walthamstow, etc., Hospital | 141 | 18 | 4 | 151 | 17 | 6 |
| Wandsworth, Bolingbroke Hospital | 357 | 10 | 0 | 326 | 5 | 0 |
| West Ham Hospital | 549 | 5 | 0 | 601 | 17 | 6 |
| West London Hospital | 1,381 | 5 | 0 | 1,276 | 17 | 6 |
| Westminster Hospital | 1,386 | 13 | 4 | 1,428 | 15 | 0 |
| **SPECIAL HOSPITALS** | | | | | | |
| **Six Chest Hospitals** | | | | | | |
| City of London Hospital for Diseases of the Chest, Victoria Park | 920 | 16 | 8 | 900 | 0 | 0 |
| Hospital for Consumption, Brompton | 3,141 | 13 | 4 | 3,420 | 0 | 0 |
| Mount Vernon Consumption Hospital, Hampstead | 1,557 | 16 | 8 | 1,530 | 0 | 0 |
| Royal Hospital for Diseases of the Chest, City Road | 601 | 5 | 0 | 613 | 2 | 6 |
| Royal National Sanatorium (Bournemouth) | 108 | 6 | 8 | 112 | 10 | 0 |
| Royal National Hospital for Consumption (Ventnor) | 270 | 16 | 8 | 337 | 10 | 0 |
| **Nineteen Children's Hospitals** | | | | | | |
| Alexandra Hospital for Hip Disease | 360 | 15 | 0 | 433 | 2 | 6 |
| Banstead Surgical Home | 39 | 0 | 0 | 39 | 7 | 6 |
| Barnet Home Hospital | 54 | 3 | 4 | 61 | 17 | 6 |
| Belgrave Hospital for Children | 329 | 6 | 8 | 225 | 0 | 0 |
| Cheyne Hospital for Incurable Children | 108 | 6 | 8 | 146 | 5 | 0 |
| East London Hospital for Children, Shadwell | 969 | 11 | 8 | 1,006 | 17 | 6 |
| Evelina Hospital for Sick Children, Southwark | Nil. | | | 225 | 0 | 0 |
| Home for Incurable Children, Hampstead | 60 | 13 | 4 | 84 | 7 | 6 |
| Home for Sick Children, Sydenham | 162 | 10 | 0 | 171 | 0 | 0 |
| Hospital for Sick Children, Great Ormond St. | 1,191 | 13 | 4 | 1,096 | 17 | 6 |
| Infants Hospital, Westminster | 160 | 8 | 8 | 112 | 10 | 0 |
| Kensington, for Children, and Dispensary | 106 | 3 | 4 | 95 | 12 | 6 |
| Paddington Green Hospital for Children | 297 | 18 | 4 | 337 | 10 | 0 |

| | Awards 1909 £ s. d. | Awards 1908 £ s. d. |
|---|---|---|
| Queen's Hospital for Children, Hackney Road | 949 0 0 | 1,001 5 0 |
| Royal Waterloo Hospital for Children and Women .. .. .. .. .. .. | 195 0 0 | 202 10 0 |
| St. Mary's Hospital, Plaistow .. .. .. | 303 6 8 | 326 5 0 |
| St. Monica's Hospital, Brondesbury .. .. | 86 13 4 | 95 12 6 |
| Victoria Hospital for Children, Chelsea .. | 845 0 0 | 843 15 0 |
| Victoria Home, Margate .. .. .. | 43 6 8 | 45 0 0 |
| Hospital for Hip Disease, Sevenoaks .. .. | 59 11 8 | 56 5 0 |
| EIGHT LYING-IN HOSPITALS | | |
| British Lying-in Hospital, Endell Street .. | 130 0 0 | 119 5 0 |
| City of London Lying-in Hospital, City Road. | 200 0 0 | 200 0 0 |
| Clapham Maternity Hospital .. .. .. | 52 0 0 | 45 0 0 |
| East End Mothers' Home .. .. .. | 91 0 0 | 84 7 6 |
| General Lying-in Hospital, Lambeth .. .. | 130 0 0 | 101 5 0 |
| Plaistow Maternity Hospital .. .. | 52 0 0 | 67 10 0 |
| Queen Charlotte's Lying-in Hospital, Marylebone Road .. .. .. .. .. | 553 11 8 | 579 7 6 |
| Home for Mothers and Babies, Woolwich .. | 80 3 4 | 45 0 0 |
| FIVE HOSPITALS FOR WOMEN | | |
| Chelsea Hospital for Women .. .. .. | 305 10 0 | 343 2 6 |
| Hospital for Women, Soho Square .. .. | 422 10 0 | 500 12 6 |
| Grosvenor Hospital for Women and Children, Vincent Square .. .. .. .. | 195 0 0 | 208 2 6 |
| New Hospital for Women, Euston Road .. | 17 6 8 | 399 7 6 |
| Samaritan Free Hospital Marylebone Road .. | 426 16 8 | 455 12 6 |
| TWENTY-THREE OTHER SPECIAL HOSPITALS | | |
| Cancer Hospital, Brompton .. .. .. | Nil. | Nil. |
| Middlesex Hospital, Cancer Wing .. .. | 257 16 8 | 292 10 0 |
| London Fever Hospital, Islington .. .. | 325 0 0 | 337 10 0 |
| Gordon Hospital for Fistula, Vauxhall Bridge Road .. .. .. .. .. .. | 47 13 4 | 50 12 6 |
| St. Mark's Hospital for Fistula, City Road .. | 228 11 8 | 292 10 0 |
| National Hospital for the Diseases of the Heart, Soho Square .. .. .. .. | 179 16 8 | 191 5 0 |
| Female Lock Hospital, Harrow Road .. | 240 10 0 | 281 5 0 |
| Hospital for Epilepsy, Paralysis and other diseases of the Nervous System, Maida Vale .. .. .. .. .. .. .. | 273 0 0 | 236 5 0 |
| National Hospital for the Paralysed and Epileptic .. .. .. .. .. .. | 1,084 8 4 | 1,091 5 0 |
| West End Hospital for Diseases of the Nervous System .. .. .. .. .. .. | 550 6 8 | 472 10 0 |
| Central London Ophthalmic Hospital, Gray's Inn Road .. .. .. .. .. .. | 167 18 4 | 196 17 6 |
| Royal Eye Hospital, St. George's Circus .. | 312 0 0 | 360 0 0 |
| Royal London Ophthalmic Hospital, CityRd. | 1,097 8 4 | 1,248 15 0 |
| Royal Westminster Ophthalmic Hospital, Charing Cross .. .. .. .. .. | 132 3 4 | 185 12 6 |
| Western Ophthalmic Hospital, Marylebone Rd. | 122 8 4 | 118 2 6 |
| Royal National Orthopædic Hospital, Great Portland Street .. .. .. .. .. | 290 6 8 | 225 0 0 |
| Royal Sea Bathing Hospital, Margate .. | 205 16 8 | 213 15 0 |
| St. John's Hospital for Diseases of the Skin.. | 81 5 0 | 56 5 0 |
| St. Peter's Hospital for Stone, Covent Garden | 62 16 8 | 135 0 0 |
| Central London Throat and Ear Hospital, Gray's Inn Road .. .. .. .. .. | 10 16 8 | 19 2 6 |
| Hospital for Diseases of the Throat, Golden Sq. | Nil. | 16 17 6 |
| London Throat Hospital, Great Portland St. | 24 18 4 | 28 2 6 |
| Royal Ear Hospital, Dean Street .. .. | 40 1 8 | 51 15 0 |
| Royal Dental Hospital of London .. .. | 355 6 8 | 416 5 0 |
| National Dental Hospital, 149 Great Portland Street .. .. .. .. .. .. | 48 15 0 | 33 15 0 |
| THIRTY-FOUR CONVALESCENT HOSPITALS. | | |
| Metro. Convalescent Institution, Walton .. | 444 3 4 | 478 2 6 |
| " " Broadstairs | 292 10 0 | 303 15 0 |
| " " Bexhill .. | 552 10 0 | 573 15 0 |
| All Saints' Convalescent Hospital, Eastbourne | 433 6 8 | 450 0 0 |
| All Saints' Convalescent Home, St. Leonards-on-Sea .. .. .. .. .. .. | 27 1 8 | 28 2 6 |
| Ascot Priory Convalescent Home .. .. | 92 1 8 | 95 12 6 |
| Brentwood Convalescent Home for Children.. | 10 16 8 | 11 5 0 |
| Charing Cross Hosp. Conv. Home, Limpsfield | 65 0 0 | 73 2 6 |
| Chelsea Hospital for Women Convalescent Home, St. Leonards .. .. .. .. | 48 15 0 | 50 12 6 |
| Metropolitan Hospital Home, Cranbrook .. | 16 5 0 | 39 7 6 |
| Deptford Medical Mission Convl. Home, Bexhill | 19 10 0 | 20 5 0 |
| Mrs. Gladstone's Convalescent Home, Mitcham | 92 1 8 | 95 12 6 |
| Friendly Societies Convalescent Home, Dover | 108 6 8 | 112 10 0 |
| Hahnemann Convalescent Home, Bournemouth | 32 10 0 | 33 15 0 |
| Hanwell Convalescent Home .. .. .. | 15 3 4 | 13 10 0 |
| Hastings, Fairlight Convalescent Home .. | 26 0 0 | 31 10 0 |
| Hendon, Ossulston Home .. .. .. .. | 71 10 0 | 64 2 6 |
| Herbert Convalescent Home, Bournemouth .. | 21 13 4 | 22 10 0 |

| | Awards 1909 £ s. d. | Awards 1908 £ s. d. |
|---|---|---|
| Herne Bay Baldwin-Brown Convalescent Home | 65 0 0 | 67 10 0 |
| Homœopathic Hospital Con. Home, Eastbourne | 10 16 8 | 16 17 6 |
| Hemel Hempstead Convalescent Home .. | 21 13 4 | 31 10 0 |
| Mrs. Kitto's Convalescent Home, Reigate .. | 54 3 4 | 56 5 0 |
| London Hospital Convalescent Home, Tankerton .. .. .. .. .. .. | 78 0 0 | 81 0 0 |
| Mary Wardell Convalescent Home for Scarlet Fever .. .. .. .. .. .. | 65 0 0 | 67 10 0 |
| Police Seaside Home, Brighton .. .. | 54 3 4 | 50 12 6 |
| St. Andrew's Convalescent Home, Clewer .. | 108 6 8 | 112 10 0 |
| St. Andrew's Convalescent Home, Folkestone | 195 0 0 | 202 10 0 |
| St. John's Home for Convalescent and Crippled Children, Brighton .. .. .. | 24 18 4 | 22 10 0 |
| St. Joseph's Convalescent Home, Bournemouth | 48 15 0 | 56 5 0 |
| St. Leonards-on-Sea Convalescent Home for Poor Children .. .. .. .. .. | 108 6 8 | 112 10 0 |
| St. Mary's Convalescent Home, Broadstairs.. | 162 10 0 | — |
| St. Mary's Convalescent Home, Shortlands .. | 24 18 4 | 25 17 6 |
| St. Michael's Convalescent Home, Westgate-on-Sea .. .. .. .. .. .. | 37 18 4 | 39 7 6 |
| Seaside Convalescent Hospital, Seaford .. | 162 10 0 | 168 15 0 |
| TWENTY-FOUR COTTAGE HOSPITALS. | | |
| Acton Cottage Hospital .. .. .. | 98 11 8 | 91 2 6 |
| Beckenham Cottage Hospital .. .. .. | 88 16 8 | 92 5 0 |
| Blackheath and Charlton Cottage Hospital .. | 86 13 4 | 90 0 0 |
| Bromley, Kent, Cottage Hospital .. .. | 137 11 8 | 137 5 0 |
| Bushey Heath Cottage Hospital .. .. | 45 10 0 | 42 15 0 |
| Canning Town Cottage Hospital .. .. | 96 8 4 | 84 7 6 |
| Chislehurst, Sidcup and Cray Valley Cottage Hospital .. .. .. .. .. .. | 73 13 4 | 73 2 6 |
| Coldash Cottage Hospital .. .. .. | 23 16 8 | 24 15 0 |
| East Ham Cottage Hospital .. .. .. | 75 16 8 | 81 0 0 |
| Eltham Cottage Hospital .. .. .. | 85 11 8 | 69 15 0 |
| Enfield Cottage Hospital .. .. .. | 76 18 4 | 73 2 6 |
| Epsom and Ewell Cottage Hospital .. .. | 50 18 4 | 45 0 0 |
| Hounslow Cottage Hospital .. .. .. | 46 11 8 | 45 0 0 |
| Livingstone Dartford Cottage Hospital .. | 99 13 4 | 108 0 0 |
| Kingston, Victoria Hospital .. .. .. | 99 13 4 | 86 12 6 |
| Mildmay Cottage Hospital .. .. .. | 43 6 8 | Nil. |
| Reigate and Redhill Cottage Hospital .. | 113 15 0 | 123 15 0 |
| Sidcup Cottage Hospital .. .. .. | 43 6 8 | 45 0 0 |
| Tilbury (Passmore Edwards) Cottage Hospital | 54 3 4 | 56 5 0 |
| Willesden Cottage Hospital .. .. .. | 123 10 0 | 123 15 0 |
| Wimbledon Cottage Hospital .. .. .. | 61 15 0 | 61 17 6 |
| Wimbledon (South) Cottage Hospital .. | 58 10 0 | 66 7 6 |
| Wood Green Cottage Hospital .. .. | 75 16 8 | 66 7 6 |
| Woolwich and Plumstead Cottage Hospital .. | 54 3 4 | 56 5 0 |
| TWELVE INSTITUTIONS. | | |
| Hospital for Invalid Gentlewomen, Harley St. | 108 6 8 | 112 10 0 |
| S. Saviour's Hospital and Nursing Home .. | 108 6 8 | 112 10 0 |
| Invalid Asylum, Stoke Newington .. .. | 37 18 4 | 39 7 6 |
| Firs Home, Bournemouth .. .. .. | 41 3 4 | 42 15 0 |
| St. Catherine's Home, Ventnor .. .. | 21 13 4 | 22 10 0 |
| Friedenheim Hospital for the Dying .. .. | 342 6 8 | 355 10 0 |
| Free Home for Dying, Clapham .. .. | 111 11 8 | 126 0 0 |
| St. Luke's House, Pembridge Square .. .. | 169 0 0 | 180 0 0 |
| Santa Claus Home, Highgate .. .. .. | 65 0 0 | 68 12 6 |
| Royal Mineral Water Hospital, Bath .. | 54 3 4 | 56 5 0 |
| Winifred House, Holloway .. .. .. | 63 18 4 | 64 2 6 |
| All Saints, Highgate .. .. .. .. | 28 3 4 | 24 15 0 |
| Sunshine Home of Recovery, Hurstpierpoint | Nil. | .. |
| FIFTY-NINE DISPENSARIES. | | |
| Battersea Provident Dispensary .. .. | 210 3 4 | 213 15 0 |
| Billingsgate Dispensary .. .. .. | 81 5 0 | 118 2 6 |
| Blackfriars Provident Dispensary .. .. | 19 10 0 | 22 10 0 |
| Bloomsbury Provident Dispensary .. .. | 16 5 0 | 16 17 6 |
| Brixton, etc., Dispensary .. .. .. | 53 1 8 | 56 5 0 |
| Brompton Provident Dispensary .. .. | 20 11 8 | 19 2 6 |
| Buxton Street Dispensary .. .. .. | 21 13 4 | 28 2 6 |
| Camberwell Provident Dispensary .. .. | 81 5 0 | 90 0 0 |
| Camden Town Provident Dispensary .. | 16 5 0 | 16 17 6 |
| Chelsea, Brompton and Belgrave Dispensary.. | 44 8 4 | 50 12 6 |
| Chelsea Provident Dispensary .. .. | 14 1 8 | 11 5 0 |
| Childs Hill Provident Dispensary .. .. | 15 3 4 | 16 17 6 |
| City Dispensary .. .. .. .. .. | 65 0 0 | 56 5 0 |
| Clapham General and Provident Dispensary.. | 24 18 4 | 28 2 6 |
| Deptford Medical Mission .. .. .. | 26 0 0 | 28 2 6 |
| Eastern Dispensary .. .. .. .. | 43 6 8 | 42 15 0 |
| East Dulwich Provident Dispensary .. .. | 57 8 4 | 54 0 0 |
| Farringdon General Dispensary .. .. | 55 5 0 | 45 0 0 |
| Finsbury Dispensary .. .. .. .. | 45 10 0 | 63 0 0 |
| Forest Hill Provident Dispensary .. .. | 40 1 8 | 42 15 0 |

| | Awards 1909 £ | s. | d. | 1908 £ | s. | d. |
|---|---|---|---|---|---|---|
| Greenwich Provident Dispensary .. .. | 36 | 16 | 8 | 39 | 7 | 6 |
| Hackney Provident Dispensary .. .. | 20 | 11 | 8 | 20 | 5 | 0 |
| Hampstead Provident Dispensary .. .. | 65 | 0 | 0 | 69 | 15 | 0 |
| Holloway and North Islington Dispensary .. | 17 | 6 | 8 | 22 | 10 | 0 |
| Islington Dispensary .. .. .. .. .. | 62 | 16 | 8 | 68 | 12 | 6 |
| Islington Medical Mission .. .. .. | 67 | 3 | 4 | 49 | 10 | 0 |
| Kennington and Vauxhall Prov. Dispensary | 15 | 3 | 4 | 15 | 15 | 0 |
| Kensal Town Provident Dispensary .. .. | 14 | 1 | 8 | 16 | 17 | 6 |
| Kentish Town Medical Mission .. .. | 22 | 15 | 0 | 23 | 12 | 6 |
| Kilburn, Maida Vale and S. John's Wood Dispensary .. .. .. .. .. .. | 37 | 18 | 4 | 45 | 0 | 0 |
| Kilburn Provident Medical Institution .. | 54 | 3 | 4 | 58 | 10 | 0 |
| London Dispensary, Spitalfields .. .. | 20 | 11 | 8 | 18 | 0 | 0 |
| London Medical Mission, Endell Street .. | 130 | 0 | 0 | 153 | 0 | 0 |
| Margaret Street, for Consumption .. .. | 33 | 11 | 8 | 34 | 17 | 6 |
| Metropolitan Dispensary .. .. .. | 67 | 3 | 4 | 74 | 5 | 0 |
| Mildmay Medical Mission Dispensary.. .. | 17 | 6 | 8 | 22 | 10 | 0 |
| Notting Hill Provident Dispensary .. .. | 19 | 10 | 0 | 11 | 5 | 0 |
| Paddington Provident Dispensary .. .. | 39 | 0 | 0 | 39 | 7 | 6 |
| Public Dispensary, Drury Lane, W.C. .. | 44 | 8 | 4 | 33 | 15 | 0 |
| Queen Adelaide's Dispensary .. .. .. | 27 | 1 | 8 | 30 | 7 | 6 |
| Royal General Dispensary .. .. .. | 28 | 3 | 4 | 30 | 7 | 6 |
| Royal Pimlico Provident Dispensary .. .. | 46 | 11 | 8 | 54 | 0 | 0 |
| Royal South London Dispensary .. .. | 50 | 18 | 4 | 51 | 15 | 0 |
| St. George's, Hanover Square, Dispensary .. | 36 | 16 | 8 | 30 | 7 | 6 |
| St. John's Wood Provident Dispensary .. | 56 | 6 | 8 | 57 | 7 | 6 |
| St. Marylebone General Dispensary .. .. | 58 | 10 | 0 | 60 | 15 | 0 |
| St. Pancras and Northern Dispensary .. | 39 | 0 | 0 | 36 | 0 | 0 |
| South Lambeth, Stockwell, and North Brixton Dispensary .. .. .. .. .. .. | 41 | 3 | 4 | 43 | 17 | 6 |
| Stamford Hill, Stoke Newington Dispensary.. | 55 | 5 | 0 | 63 | 0 | 0 |
| Tower Hamlets Dispensary .. .. .. | 50 | 18 | 4 | 56 | 5 | 0 |
| Walworth Provident Dispensary .. .. | 16 | 5 | 0 | 18 | 0 | 0 |
| Wandsworth Common Provident Dispensary | 15 | 3 | 4 | 16 | 17 | 6 |
| Westbourne Provident Dispensary .. .. | 19 | 10 | 0 | 22 | 10 | 0 |
| Western Dispensary .. .. .. .. | 80 | 3 | 4 | 76 | 10 | 0 |

| | Awards 1909 £ | s. | d. | 1908 £ | s. | d. |
|---|---|---|---|---|---|---|
| Western General Dispensary .. .. .. .. | 109 | 8 | 4 | 113 | 12 | 6 |
| West Ham Provident Dispensary .. .. | 13 | 0 | 0 | 14 | 12 | 6 |
| Westminster General Dispensary .. .. | 48 | 15 | 0 | 51 | 15 | 0 |
| Whitechapel Provident Dispensary .. .. | 37 | 18 | 4 | 38 | 5 | 0 |
| Woolwich Provident Dispensary .. .. | 41 | 3 | 4 | 38 | 5 | 0 |
| THIRTY NURSING ASSOCIATIONS. | | | | | | |
| Belvedere, Abbey Wood .. .. .. .. | 8 | 5 | 0 | 8 | 11 | 0 |
| Brixton .. .. .. .. .. .. .. .. | 28 | 0 | 0 | 27 | 6 | 0 |
| Central St. Pancras .. .. .. .. .. | 21 | 0 | 0 | 20 | 10 | 0 |
| Chelsea and Pimlico .. .. .. .. .. | 28 | 0 | 0 | 27 | 6 | 0 |
| Hackney .. .. .. .. .. .. .. .. | 33 | 1 | 0 | 34 | 4 | 0 |
| Hammersmith .. .. .. .. .. .. .. | 49 | 0 | 0 | 54 | 14 | 0 |
| Hampstead .. .. .. .. .. .. .. | 21 | 0 | 0 | 20 | 10 | 0 |
| Isleworth .. .. .. .. .. .. .. | 8 | 5 | 0 | 13 | 13 | 0 |
| Kensington .. .. .. .. .. .. .. | 49 | 0 | 0 | 61 | 10 | 0 |
| Kilburn .. .. .. .. .. .. .. .. | 8 | 5 | 0 | 8 | 11 | 0 |
| Kingston .. .. .. .. .. .. .. | 35 | 0 | 0 | 34 | 4 | 0 |
| Metropolitan (Bloomsbury) .. .. .. .. | 56 | 0 | 0 | 68 | 6 | 0 |
| St. Olave's (Bermondsey) .. .. .. .. | 28 | 0 | 0 | 47 | 16 | 0 |
| Paddington and Marylebone .. .. .. | 49 | 0 | 0 | 47 | 16 | 0 |
| Peckham .. .. .. .. .. .. .. .. | 8 | 5 | 0 | .. | | |
| Plaistow .. .. .. .. .. .. .. .. | 78 | 10 | 0 | 68 | 8 | 0 |
| „ (Maternity) .. .. .. .. .. | 103 | 5 | 0 | 150 | 6 | 0 |
| Rotherhithe .. .. .. .. .. .. .. | 8 | 5 | 0 | 13 | 13 | 0 |
| Shoreditch .. .. .. .. .. .. .. | 63 | 0 | 0 | 88 | 16 | 0 |
| Silvertown .. .. .. .. .. .. .. | 12 | 8 | 0 | 12 | 16 | 0 |
| South London (Battersea) .. .. .. .. | 63 | 0 | 0 | 61 | 10 | 0 |
| Southwark .. .. .. .. .. .. .. | 42 | 0 | 0 | 41 | 0 | 0 |
| South Wimbledon .. .. .. .. .. .. | 20 | 13 | 0 | 21 | 7 | 0 |
| Tottenham .. .. .. .. .. .. .. | 7 | 0 | 0 | 6 | 16 | 0 |
| Westminster .. .. .. .. .. .. .. | 28 | 0 | 0 | 27 | 6 | 0 |
| Woolwich .. .. .. .. .. .. .. | 28 | 18 | 0 | 29 | 18 | 0 |
| East London .. .. .. .. .. .. .. | 182 | 0 | 0 | 177 | 13 | 0 |
| North London .. .. .. .. .. .. .. | 70 | 0 | 0 | 75 | 3 | 0 |
| London District .. .. .. .. .. .. | 525 | 0 | 0 | 492 | 0 | 0 |
| Sick-Room Helps Society .. .. .. .. | 14 | 0 | 0 | 8 | 11 | 0 |

